# Metabolic activity, urease production, antibiotic resistance and virulence in dual species biofilms of *Staphylococcus epidermidis* and *Staphylococcus aureus*

**DOI:** 10.1371/journal.pone.0172700

**Published:** 2017-03-06

**Authors:** Ilse Vandecandelaere, Filip Van Nieuwerburgh, Dieter Deforce, Tom Coenye

**Affiliations:** 1 Laboratorium voor Farmaceutische Microbiologie, Universiteit Gent, Ottergemsesteenweg, Gent, Belgium; 2 Laboratorium voor Farmaceutische Biotechnologie, Universiteit Gent, Ottergemsesteenweg, Gent, Belgium; Universitatsklinikum Hamburg-Eppendorf, GERMANY

## Abstract

In this paper, the metabolic activity in single and dual species biofilms of *Staphylococcus epidermidis* and *Staphylococcus aureus* isolates was investigated. Our results demonstrated that there was less metabolic activity in dual species biofilms compared to *S*. *aureus* biofilms. However, this was not observed if *S*. *aureus* and *S*. *epidermidis* were obtained from the same sample. The largest effect on metabolic activity was observed in biofilms of *S*. *aureus* Mu50 and *S*. *epidermidis* ET-024. A transcriptomic analysis of these dual species biofilms showed that urease genes and genes encoding proteins involved in metabolism were downregulated in comparison to monospecies biofilms. These results were subsequently confirmed by phenotypic assays. As metabolic activity is related to acid production, the pH in dual species biofilms was slightly higher compared to *S*. *aureus* Mu50 biofilms. Our results showed that *S*. *epidermidis* ET-024 in dual species biofilms inhibits metabolic activity of *S*. *aureus* Mu50, leading to less acid production. As a consequence, less urease activity is required to compensate for low pH. Importantly, this effect was biofilm-specific. Also *S*. *aureus* Mu50 genes encoding virulence-associated proteins (Spa, SplF and Dps) were upregulated in dual species biofilms compared to monospecies biofilms and using *Caenorhabditis elegans* infection assays, we demonstrated that more nematodes survived when co-infected with *S*. *epidermidis* ET-024 and *S*. *aureus* mutants lacking functional *spa*, *splF* or *dps* genes, compared to nematodes infected with *S*. *epidermidis* ET-024 and wild- type *S*. *aureus*. Finally, *S*. *epidermidis* ET-024 genes encoding resistance to oxacillin, erythromycin and tobramycin were upregulated in dual species biofilms and increased resistance was subsequently confirmed. Our data indicate that both species in dual species biofilms of *S*. *epidermidis* and *S*. *aureus* influence each other’s behavior, but additional studies are required necessary to elucidate the exact mechanism(s) involved.

## Introduction

*Staphylococcus aureus* is an important nosocomial pathogen [1] and infections caused by *S*. *aureus* range from skin infections to systemic infections [2). The success of *S*. *aureus* as a pathogen can partially be attributed to its antibiotic resistance and to its ability to form biofilms [3–5]. Also, the production of a wide range of virulence factors enables *S*. *aureus* to cause infections [4,6,7]. For instance, *Staphylococcus* protein A (SpA) contributes to the onset and severity of *S*. *aureus* infections [8–11]. Exoenzymes (including SplF proteases) can degrade host proteins or interfere with host signaling cascades [6,12] while Dps proteins (homologs of MrgA in *Bacillus subtilis*) play a role in infections by conferring resistance to nitric oxide and hydrogen peroxide [13,14]. These Dps proteins are usually described as Dps homologues to the *Escherichia coli* Dps proteins. In *S*. *aureus* this homologue has originally been described as MrgA (Metallo regulon gene A) in analogy to *Bacillus subtilis* [15].

*Staphylococcus epidermidis* is a commensal organism but can also cause device-related infections [2,16,17]. The ability of *S*. *epidermidis* to form biofilms is an important virulence factor [18]. Furthermore, *S*. *epidermidis* can act as a reservoir of antibiotic resistance genes for *S*. *aureus* [19,20]. A large fraction of clinical *S*. *epidermidis* isolates showed resistance to different antibiotics including β-lactams (e.g. methicillin resistance encoded by *mecA*), macrolides (e.g. erythromycin resistance encoded by *ermABC*) and aminoglycosides (e.g. tobramycin resistance encoded by *aacA*-*aphD*) [21–23].

The role of multispecies biofilms in device-related infections is not yet completely understood but several papers already reported that *S*. *aureus* and *S*. *epidermidis* are often co-isolated from biofilms present on indwelling medical devices [24–26].

Several studies focused on the gene expression in *S*. *aureus* biofilms. For instance, Resch *et al*. (2005) compared gene expression levels of planktonic *S*. *aureus* cultures to those in biofilms. Urease genes and genes encoding proteins involved in formate synthesis were upregulated in biofilms compared to planktonic cells and it was suggested that urease activity protected the cells by counteracting the low pH (resulting from formate metabolism) [5]. In general, urease activity protects bacteria in acidic environments by neutralizing acids [27]. For instance, it was already reported that treating *S*. *aureus* cultures with acids resulted in an upregulation of urease activity [28]. Urease (urea amidohydrolase) catalyzes the hydrolysis of urea, leading to two molecules of ammonia and one molecule of carbon dioxide in aqueous environments [29,30]. Urease enzymes are structurally complex, containing an apoenzyme (α, β and γsubunits encoded by *ureABC*) and accessory proteins (encoded by *ureDEFG*) [26,29,30].

In the present study, we have determined the metabolic activity in single and dual species biofilms formed by various *S*. *aureus* and *S*. *epidermidis* strains. Subsequently, we performed a transcriptome analysis of single and dual species biofilms of *S*. *epidermidis* ET-024 and *S*. *aureus* Mu50 (the pair of strains for which the largest effect on metabolic activity was observed). We then set out experiments to confirm several of the observations made during the transcriptomic analysis.

## Materials and methods

### Strains

*S*. *epidermidis* ET-024 was isolated from an endotracheal tube (ET) biofilm of a mechanically ventilated patient [24] while *S*. *aureus* Mu50 was isolated from an infected wound of an infant [31]. *S*. *aureus* ET-058 and *S*. *epidermidis* ET-059 were isolated from the same ET biofilm, as were *S*. *aureus* ET-106/*S*. *epidermidis* ET-107, *S*. *aureus* ET-131/*S*. *epidermidis* ET-130 and *S*. *aureus* ET-181/*S*. *epidermidis* ET-167 [32]. Also, five additional *S*. *aureus* reference strains were included: *S*. *aureus* JE2, *S*. *aureus* LMG 8224, *S*. *aureus* ATCC 6538P, *S*. *aureus* LMG 10147 and *S*. *aureus* Newbould305 [33–36]. Pure cultures were made on Mueller Hinton Agar (MHA; 24 hours at 37°C). *S*. *aureus* JE2 transposon mutants (NARSA; Network on Antimicrobial Resistance of *Staphylococcus aureus*) lacking functional *spa* (NE286), *splF* (NE1764) or *dps* (NE1929) genes were used to infect *C*. *elegans*. These isolates were grown (24 hours, 37°C) on MHA containing 5 μg/ml erythromycin [37]. All strains were stored at -80°C using the MicroBank System (Pro-Lab Diagnostics, Neston, UK).

### Biofilm formation

Overnight cultures of *S*. *epidermidis* ET-024 and *S*. *aureus* Mu50 were diluted to approx. 5 x 10^7^ colony forming units (CFU)/ml (for monospecies biofilms) or to approx. 10^8^ CFU/ml (for dual species biofilms; equal volumes of both cultures were subsequently mixed). For planktonic cultures, 5 ml of diluted cell suspensions was transferred to falcon tubes and incubated in a shaking warm water bath (24 hours, 37°C).

For biofilm formation we used the approach described by Peeters *et al*. (2008) [38] and 100 μl of diluted cells suspensions were transferred to the wells of a 96 well microtiter plate (MTP); two rows served as blanks. After 4 hours at 37°C, all wells were rinsed with 100 μl of physiological saline (PS; 0.9% NaCl) and 100 μl of MHB was added (20 additional hours of incubation at 37°C). In all dual species biofilms experiments described in this study, we have used 1:1 ratios of *S*. *aureus* and *S*. *epidermidis* strains. In order to test the relative fitness of *S*. *aureus* and *S*. *epidermidis*, we have also performed biofilm experiments with different ratios of *S*. *aureus* Mu50 and *S*. *epidermidis* ET-024 cells (i.e. 1/1, 1/10 and 10/1). After incubation, cells were removed from the wells by two rounds of sonication (5 min) and vortexing (600 rpm; 5 min). Serial dilutions (10^−1^ to 10^−8^) were made and plated on MHA (24 hours at 37°C). After counting the colonies, the number (log_10_) of CFU per biofilm was determined. Also, the biofilm forming capacities of strains investigated in this study were evaluated by a crystal violet assay.

The pH of single and dual species biofilms of *S*. *epidermidis* ET-024 and *S*. *aureus* Mu50 was measured after 24 hours incubation at 37°C. To do this, biofilms were grown in a 24 well MTP (1 ml of cell suspension per well) and after 4 hours of incubation, the wells were rinsed and 1 ml of MHB was added (20 additional hours of incubation at 37°C).

### Determination of metabolic activity

The metabolic activity of *S*. *aureus* and *S*. *epidermidis* in single and dual species biofilms (96 well MTP) was determined by a resazurin-based assay. After incubation, biofilms were rinsed and 120 μl of a resazurin solution (Promega, Leiden, The Netherlands) was added to each well. The MTP was incubated for 20 min at 37°C and afterwards, the fluorescence (excitation wavelength: 560 nm; emission wavelength: 590 nm) was measured (Envision, PerkinElmer, Zaventem, Belgium). The values were normalized to the number (log_10_) of CFU per biofilm.

### pH measurements of single and dual species biofilms of *S*. *epidermidis* ET-024 and *S*. *aureus* Mu50

After incubation, the biofilm supernatant was collected and subsequently centrifuged to remove all cells. The obtained supernatant was again added to the biofilms and the biofilm cells were suspended. The pH of these suspensions was measured (3 biological replicates) using a pH probe (Hanna Instruments, Temse, Belgium).

### RNA extraction of single and dual species cultures of *S*. *epidermidis* ET-024 and *S*. *aureus* Mu50

Three biological replicates were included. Planktonic cultures of *S*. *aureus* Mu50 and *S*. *epidermidis* ET-024 were centrifuged for 5 min at 5000 rpm and the cell pellets were rinsed with 5 ml RNA*later* (Ambion, Life Technologies, Ledeberg, Belgium). For the biofilms, all wells of the MTP were rinsed with 100 μl of RNA*later*. Afterwards, 100 μl of RNA*later* was added to all wells and the cells were removed from the wells as described above. Subsequently, biofilm cells were collected in a falcon tube and this procedure was repeated. Thereafter, the tubes were centrifuged for 5 min at 5000 rpm and the cell pellet was rinsed with 5 ml of RNA*later*. The RiboPure RNA purification Bacteria kit (Ambion, Life Technologies) was used according to the manufacturer’s instructions and the RNA solution was stored at -80°C.

### RNA-Seq on single and dual species biofilms of *S*. *epidermidis* ET-024 and *S*. *aureus* Mu50

Three biological replicates per sample were sequenced. Approximately 30 ng of rRNA depleted RNA was used to create barcoded strand specific cDNA sequencing libraries with TruSeq stranded library preparation kit (Illumina, San Diego, CA, USA). The libraries were equimolarly pooled and sequenced using a HiSeq (Illumina), generating unpaired reads of 100 bp. After sequencing, the data were demultiplexed and the CLC Genomic Workbench 8.5.1 (Qiagen, Aarhus, Denmark) was used for analysis.

Initial quality control (based on length and Phred scores) resulted in more than 10,000,000 reads per sample. Reads were mapped to *S*. *aureus* Mu50 [30] and *S*. *epidermidis* ET-024 genomes [39] using stringent mapping conditions i.e. reads must map to the entire gene sequence with 100% similarity and no mapping to the flanking regions was allowed. Reads per kb per million (RPKM) expression values were calculated and the p value was determined using edgeR statistics. The full transcriptomic dataset was deposited in the Gene Expression Omnibus (www.ncbi.nlm.nih.gov/geo)—accession number GSE 79575.

### qPCR analysis of urease coding genes

Prior to qPCR, RNA was converted to cDNA using the qScript cDNA SuperMix (Quanta BioSciences, Beverly, MA, USA) using the manufacturer’s instructions.

Species-specific primers targeting the urease genes of *S*. *aureus* Mu50 and *S*. *epidermidis* ET-024 were developed S1 File). The expression values of *S*. *aureus* Mu50 and *S*. *epidermidis* ET-024 urease genes were normalized by the expression values of selected reference genes. We used publically available primer sequences [40] and newly designed species-specific primers (Table A in S1 File).

qPCR reactions were carried out using the Bio-Rad real time PCR detection CFX-96 apparatus (Bio-Rad) and PerfeCTa SYBR Green FastMix (Quanta BioSciences) according the manufacturer’s instructions. Following PCR, the melting curves were determined by increasing the temperature from 65°C to 95°C in steps of 0.5°C (each for 5 s).

### Determination of urease activity

Urease activity in single and dual species biofilms was measured using the urease activity assay kit (Sigma-Aldrich, Bornem, Belgium). Biofilms were grown in a 96 well MTP and afterwards, biofilm cell suspensions were collected. After centrifugation at 13000 rpm for 5 min, cell pellets were resuspended in assay buffer and urease activity was determined according to the manufacturer’s instructions. The absorbance at 670 nm (A_670_) was measured (Tecan, Männedorf, Switzerland) and the urease activity (units/L) was calculated according to the manufacturer’s instructions. The values were normalized to the number (log_10_) of CFU per biofilm.

### Exposing biofilms to antibiotics

Minimal inhibitory concentrations (MIC) of oxacillin, erythromycin and tobramycin for *S*. *epidermidis* ET-024 and *S*. *aureus* Mu50 were determined according to the CLSI guidelines and *S*. *aureus* LMG 10147 was included as a reference strain.

After 4 hours of incubation, single and dual species biofilms were exposed to 4 μg/ml of oxacillin, 2 μg/ml of erythromycin and 16 μg/ml of tobramycin. The plates were incubated for 20 additional hours at 37°C and afterwards, serial dilutions (10^−1^ to 10^−8^) were made and inoculated on MHA supplemented with oxacillin (4 μg/ml), erythromycin (2 μg/ml) or tobramycin (16 μg/ml). Plates were incubated for at least 24 hours (37°C) and afterwards, the number (log_10_) of CFU of *S*. *aureus* Mu50 and *S*. *epidermidis* ET-024 per biofilm was determined.

### *C*. *elegans* infection assay

*C*. *elegans* strain N2 (of which *glp-4* and *sek-1* genes were deleted) was used [41, 42] and worms were grown as previously described [39].

The effect on survival of *C*. *elegans* after infection with *S*. *epidermidis* ET-024, *S*. *aureus* Mu50, *S*. *aureus* JE2 and NARSA mutants lacking a functional *spa* (NE286), *splF* (NE1764) or *dps* (NE1929) gene, was evaluated. Prior to the actual experiments, the *C*. *elegans* assay was validated and found to be a good system to test virulence of the NARSA mutants (S1 File). The infection assay was performed in a 96 well MTP and approx. 30 L4 stage worms were added per well in medium containing 95% M9 buffer (3 g KH_2_PO_4_, 6 g Na_2_HPO_4_, 1 ml 1 M MgSO_4_ per liter), 5% Brain Heart Infusion Broth (Lab M Limited, Heywood, UK) and 0.1 v/v % of a 5 mg/ml cholesterol solution (Sigma-Aldrich). Worms were infected by adding 25 μl of a bacterial cell suspension (approx. 10^9^ CFU/ml). Medium was added to a final volume of 100 μl per well. The MTP was incubated (25°C) and scored for live and dead worms after 48 hours of infection (post infection; p.i.).

The number (log_10_) of CFU per nematode was determined as described by Brackman *et al*. [43] and the dilutions were inoculated on MHA supplemented with 7.5% NaCl. Plates were incubated (37°C) for at least 24 hours and subsequently, the number (log_10_) of CFU per worm was determined.

### Statistical analysis

Statistical analysis was performed using the Mann-Whitney U test or the Kruskall-Walis test (SPSS, version 23.0, Chicago, IL, USA). p values smaller than 0.01 were considered as statistically significant.

## Results

### Metabolic activity in single and dual species biofilms

In order to test the relative fitness, we have performed biofilm experiments with different ratios of *S*. *aureus* Mu50 and *S*. *epidermidis* ET-024 (i.e. 1/1; 1/10 and 10/1). The results showed that the initial ratios of both species are approx. maintained throughout the experiment. So, there is no effect of one species on the survival of the other species. Also, the results of the crystal violet assay demonstrated that all strains were able to form biofilms; the strongest biofilm forming strains are the *S*. *aureus* reference strains (i.e. Mu50, LMG 8224, LMG 10147, Newbould and ATCC 6538P) (Table C in S1 File).

The metabolic activity in single and dual species biofilms of *S*. *aureus* and *S*. *epidermidis* isolates was normalized to the number of CFU per biofilm (see Table B in S1 File).

**Fig 1:**
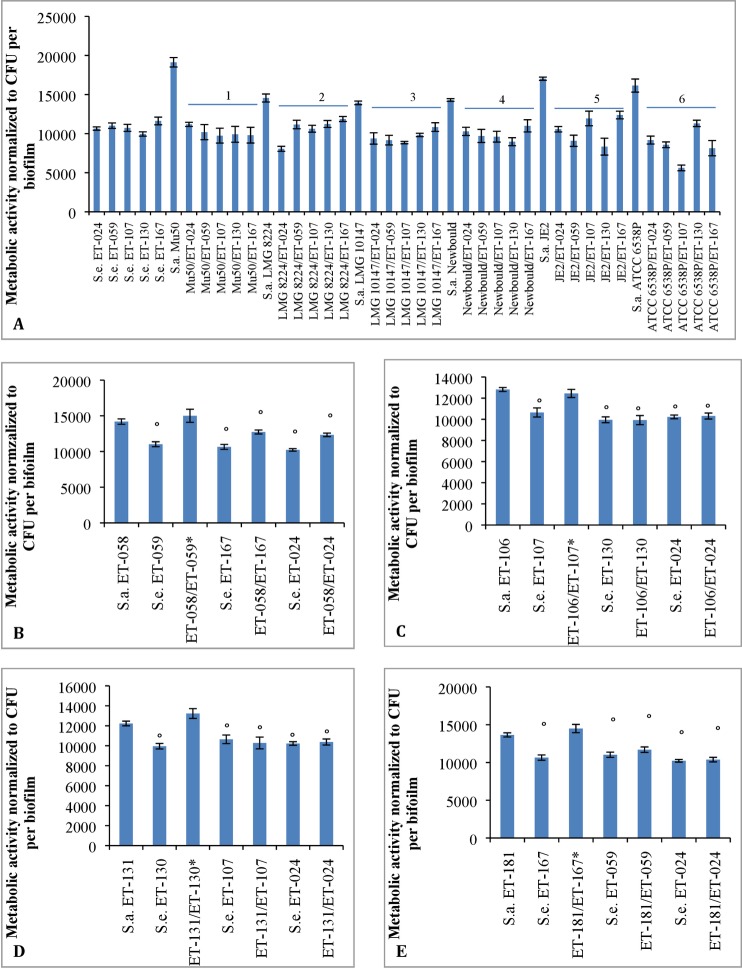
The metabolic activity in single and dual species biofilms of *S*. *aureus* and *S*. *epidermidis*. The fluorescence is normalized to the number (log_10_)of CFU per biofilm. Three biological replicates were included and each experiment was repeated at least 6 times per biological replicate. Standard error mean (SEM; error bars) was calculated for all conditions. **A**: The metabolic activity in single and dual species biofilms formed by *S*. *epidermidis* ET isolates (ET-024, ET-059, ET-107, ET-130 and ET-167) and *S*. *aureus* reference strains (Mu50, LMG 8224, LMG 10147, Newbould605 and ATCC 6568P). 1: p < 0.01, compared to *S*. *aureus* Mu50; 2: p < 0.01, compared to *S*. *aureus* LMG 8224; 3: p < 0.01, compared to *S*. *aureus* LMG 10147; 4: p < 0.01, compared to *S*. *aureus* Newbould; 5: p < 0.01, compared to *S*. *aureus* JE2; 6: p < 0.01, compared to *S*. *aureus* ATCC 6538P. **B-E**: The metabolic activity in single and dual species biofilms formed by *S*. *aureus* ET isolates (ET-058, ET-106, ET-131 and ET-181), *S*. *epidermidis* ET isolates (ET-024, ET-059, ET-107, ET-130 and ET-167). *: both *S*. *epidermidis* and *S*. *aureus* isolates were obtained from the same ET biofilm. °; p < 0.01, compared to *S*. *aureus* ET-058 (Fig 1B), *S*. *aureus* ET-106 (Fig 1C), *S*. *aureus* ET-131 (Fig 1D) and *S*. *aureus* ET-181 (Fig 1E), respectively.

The metabolic activity in dual species biofilms of *S*. *epidermidis* ET isolates (i.e. ET-024, ET-059, ET-107, ET-130 and ET-167) and *S*. *aureus* reference strains (i.e. Mu50, LMG 8224, LMG 10147, Newbould 305, JE2 and ATCC 6538P) was significantly lower than the metabolic activity in monospecies biofilms formed by these *S*. *aureus* reference strains (Fig 1A). However, the metabolic activity of these dual species biofilms is similar (p > 0.01) to the metabolic activity in biofilms formed by *S*. *epidermidis* ET isolates alone (Fig 1A).

We also investigated the metabolic activity in single and dual species biofilms formed by *S*. *aureus* and *S*. *epidermidis* isolates both obtained from ET biofilms. We observed that the metabolic activity of dual species biofilms formed by *S*. *aureus* ET isolates (i.e. ET-058, ET-106, ET-131 and ET-181) and *S*. *epidermidis* ET isolates (i.e. ET-024, ET-059, ET-107, ET-130 and ET-167) is significantly lower than the metabolic activity in monospecies biofilms formed by *S*. *aureus* ET isolates (Fig 1B–1E). However, this effect was not observed if *S*. *aureus* and *S*. *epidermidis* were obtained from the same ET biofilm (Fig 1B–1E), e.g. the metabolic activity in dual species biofilms formed by *S*. *aureus* ET-058 and *S*. *epidermidis* ET-059 was similar to the metabolic activity in *S*. *aureus* ET-058 biofilms. Also, the metabolic activity in dual species biofilms of *S*. *aureus* and *S*. *epidermidis* ET isolates is similar to the metabolic activity in monospecies biofilms formed by these *S*. *epidermidis* isolates.

Based on these results, we decided to perform a transcriptome analysis (using RNA-Seq and qPCR) on single and dual species biofilms of *S*. *aureus* Mu50 and *S*. *epidermidis* ET-024, the pair for which the largest effect on metabolic activity was observed (Fig 1A).

### Measuring the pH of single and dual species biofilms of *S*. *epidermidis* ET-024 and *S*. *aureus* Mu50

At the start of incubation, the pH of the diluted cell suspensions used for biofilm formation of *S*. *aureus* Mu50, *S*. *epidermidis* ET-024, and the dual species biofilm was very similar: 7.12 ± 0.02 (dual species biofilms), 7.20 ± 0.01 (*S*. *epidermidis* ET-024 biofilms) and 7.17 ± 0.03 (*S*. *aureus* Mu50 biofilms). After 24 hours of incubation, the pH of *S*. *aureus* Mu50 biofilms was significantly (p < 0.01) lower (6.61 ± 0.01) than the pH of *S*. *epidermidis* ET-024 biofilms (7.12 ± 0.02) and that of dual species biofilms (7.09 ± 0.02).

### Transcriptomic analysis

The results of the RNA sequencing showed that 89 genes (59 of *S*. *aureus* Mu50 and 30 of *S*. *epidermidis* ET-024) were significantly (p < 0.01) downregulated in dual species biofilms compared to monospecies biofilms (with the fold change being at least two). In contrast, 134 genes (51 of *S*. *aureus* Mu50 and 83 of *S*. *epidermidis* ET-024) were significantly upregulated in dual species biofilms compared to monospecies biofilms (with the fold change being at least two). In the present study, we focused on genes encoding proteins involved in biofilm formation, antibiotic resistance, virulence, urease activity, transport, translation and general metabolism of *S*. *aureus* Mu50 and *S*. *epidermidis* ET-024 (Table 1).

**Table 1:** List of selected *S*. *epidermidis* ET-024 and *S*. *aureus* Mu50 genes which are differentially (indicated by “fold change”) expressed in dual species biofilms compared to monospecies biofilms.

	Gene	Locus_tag	Gene function	Fold change	p *
***S*. *aureus* Mu50**					
	**Virulence-associated**				
	*spa*	SAV_RS00690	Immunoglobulin G binding protein A	3.92 + 0.21	0.00
	*dps*	SAV_RS11695	Dps general stress protein 20U	2.39 + 0.18	0.01
	*splF*	SAV_RS09715	Serine like protease F	2.38 + 0.22	0.00
	**Urease**				
	*ureA*	SAV_RS12475	Urease subunit gamma	-2.59 + 0.09	0.00
	*ureB*	SAV_RS12480	Urease subunit beta	-2.61 + 0.05	0.00
	*ureC*	SAV_RS12485	Urease subunit alpha	-3.98 + 0.08	0.00
	*ureD*	SAV_RS12505	Urease accessory protein UreD	-5.09 + 0.11	0.00
	*ureE*	SAV_RS12490	Urease accessory protein UreE	-5.97 + 0.08	0.00
	*ureF*	SAV_RS12495	Urease accessory protein UreF	-2.00 + 0.07	0.01
	*ureG*	SAV_RS12500	Urease accessory protein UreG	-2.00 +0.19	0.01
	**Translation**				
	SAVrRNA11	SAV_RS11260	5S ribosomal RNA	-2.91 + 0.27	0.00
	SAVrRNA03	SAV_RS02630	5S ribosomal RNA	-2.94 + 0.27	0.00
	SAVrRNA04	SAV_RS02845	5S ribosomal RNA	-3.93 + 0.17	0.00
	SAVrRNA14	SAV_RS11835	5S ribosomal RNA	-4.37 + 0.18	0.00
	SerS	SAV_RS00190	Seryl-tRNA synthase	-2.00 + 0.19	0.00
	**Transporters**				
	SAV1604	SAV_RS08625	Transmembrane transport protein	-2.48 + 0.07	0.00
	*lacF*	SAV_RS11985	PTS system lactose-specific transporter subunit IIA	-2.62 + 0.23	0.01
	*kdpA*	SAV_RS11365	Potassium-transporting ATPase subunit A	-2.74 + 0.22	0.00
	SAV0330	SAV_RS01835	Sugar-specific PTS component EIIB	-2.96 + 0.08	0.00
	SAV2253	SAV_RS12290	Xanthine/uracil permease family protein	-2.00 + 0.14	0.00
	*ulaA*	SAV_RS01830	PTS system ascorbate-specific transporter subunit IIC	-2.86 + 0.05	0.00
	*kdpB* (SCCmec)	SAV_RS00495	Potassium-transporting ATPase subunit B	-3.19 + 0.23	0.00
	truncated-*kdpA*	SAV_RS00490	Potassium-transporting ATPase subunit A	-3.25 + 0.20	0.00
	**Metabolism**				
	*lctE*	SAV_RS01365	L-lactate dehydrogenase	-2.16 + 0.20	0.00
	SAV1606	SAV_RS08635	Acetyl-CoA carboxylase	-2.17 + 0.01	0.00
	*lacD*	SAV_RS11990	Tagatose 1,6-diphosphate aldolase	-2.21 + 0.19	
	*lacC*	SAV_RS11995	Tagatose-6-phosphate kinase	-2.41 + 0.20	0.00
	*lacB*	SAV_RS12000	Galactose-6-phosphate isomerase subunit LacB	-2.80 + 0.25	0.00
	*pflB*	SAV_RS01275	Formate acetyltransferase	-2.61 + 0.08	0.00
	*purD*	SAV_RS05790	Phosphoribosylamine-glycine ligase	-2.93 + 0.13	0.00
	*purC*	SAV_RS05750	Phosphoribosylaminoimidazole-succinocarboxamide synthase	-2.97 + 0.17	0.00
	*purK*	SAV_RS05745	Phosphoribosylaminoimidazole carboxylase ATPase subunit	-2.98 + 0.16	0.00
	*purH*	SAV_RS05785	Bifunctional phosphoribosylaminoimidazolecarboxamide formyltransferase/IMP cyclohydrolase	-3.83 + 0.12	0.00
	*purF*	SAV_RS05770	Amidophosphoribosyltransferase	-3.91 + 0.15	0.00
	*purM*	SAV_RS05775	Phosphoribosylaminoimidazole synthetase	-4.08 + 0.13	0.00
	SAV1064	SAV_RS05740	Phosphoribosylaminoimidazole carboxylase	-3.72 + 0.18	0.00
	*purQ*	SAV_RS05760	Phosphoribosylformylglycinamidine synthase I	-4.59 + 0.13	0.00
	*purL*	SAV_RS05765	Phosphoribosylformylglycinamidine synthase II	-4.78 + 0.12	0.00
	*purN*	SAV_RS05780	Phosphoribosylglycinamidine formyltransferase	-4.81 + 0.11	0.00
	*arcA*	SAV_RS14400	Arginine deiminase	-3.10 + 0.08	0.00
	**Various**				
	SAV2401	SAV_RS13070	NirR protein	-3.81 + 0.07	0.00
***S*. *epidermidis* ET-024**					
	**Antibiotic resistance**				
	*aacA*	SERP-RS07950	6’aminoglycoside N-acetyltransferase	104.22 + 10.37	0.00
	*ermA*-1	SERP_RS06035	Erythromycine resistance	269.53 + 25.11	0.00
	*ermA*-2	SERP_RS06625	Erythromycine resistance	219.69 + 20.55	0.00
	*ermA*-3	SERP_RS12280	Erythromycine resistance	240.62 + 21.19	0.00
	*mecA*	SERP_RS12330	Penicillin-binding protein 2’	60.54 + 5.64	0.00
	**Urease**				
	*ureA*	SERP_RS09370	Urease subunit gamma	-2.55 + 0.41	0.01
	*ureB*	SERP_RS09375	Urease subunit beta	-2.59 + 0.46	0.01
	*ureC*	SERP_RS09380	Urease subunit alpha	-2.65 + 0.40	0.01
	*ureD*	SERP_RS09400	Urease accessory protein UreD	-2.16 + 0.13	0.01
	*ureE*	SERP_RS09385	Urease accessory protein UreE	-2.30 + 0.26	0.01
	*ureF*	SERP_RS09390	Urease accessory protein UreF	-2.20 + 0.16	0.01
	**Transporters**				
	*lacE*	SERP_RS08985	PTS system, lactose-specific IIBC components	-2.45 + 0.18	0.00
	*lacF*	SERP_RS08990	PTS system, lactose-specific IIA component	-3.86 + 0.14	0.00
	*arcD*	SERP_RS11080	Arginine/ornithine antiporter	-2.55 + 0.19	0.00
	**Metabolism**				
	*lacA*	SERP_RS09010	Galactose-6-phosphate isomerase subunit LacA	-3.35 + 0.17	0.00
	*lacB*	SERP_RS09005	Galactose-6-phosphate isomerase subunit LacB	-2.82 + 0.17	0.00
	*lacC*	SERP_RS09000	Tagatose-6-phosphate kinase	-2.91 + 0.18	0.00
	*lacD*	SERP_RS08995	Tagatose-1,6-diphosphate aldolase	-3.16 + 0.16	0.00
	**Various**				
	SERP0736	SERP_RS03730	Phenol soluble modulin beta 1	-2.89 + 0.17	0.00
	SERP0737	SERP_RS03735	Phenol soluble modulin beta 1	-2.69 + 0.20	0.00
	SERP0738	SERP_RS03740	Phenol soluble modulin beta 1	-4.38 + 0.22	0.00
	SERP0739	SERP_RS03745	Phenol soluble modulin beta 1	-2.31 + 0.21	0.00
	SERP0371	SERP_RS01980	exsD protein	-2.72 + 0.12	0.01
	SERP0996	SERP_RS04970	Carboxyl-terminal protease	-2.29 + 0.17	0.00
	*sspB*	SERP_RS11735	Cysteine protease precursor SspB	-2.72 + 0.06	0.00
	*sspC*	SERP_RS11740	sspC protein	-3.25 + 0.08	0.00

-: the minus sign indicates that there is a fold decrease

*: the p value was calculated using edgeR statistics (CLC Genomics Workbench 8.5.1) and statistical significance was defined as a p value smaller than 0.01. Three biological repeats were incorporated in the RNA-Seq experiment.

For both *S*. *aureus* Mu50 and *S*. *epidermidis* ET-024, genes involved in PIA production (*icaABCD*) were expressed (but no significant differences were observed between gene expression levels in dual species biofilms compared to monospecies biofilms). Also, genes known to be involved in PIA-independent biofilm formation (i.e. protein adhesion-associated biofilm formation) were expressed by both *S*. *aureus* Mu50 (e.g. *bap*) and *S*. *epidermidis* ET-024 (e.g. *aap*, *bhp*), but no significant differences in gene expression levels were observed when dual species biofilms were compared to monospecies biofilms. To investigate the potential role of eDNA production in biofilm formation, we took a closer look at the expression of *lrgAB* and *atlE* genes–while these genes are expressed, no significant differences in gene expression levels were observed in dual species biofilms compared to single species biofilms. However, in order to decipher the exact mechanism of single and dual biofilm formation of *S*. *aureus* Mu50 and *S*. *epidermidis* ET-024, more profound analyses are indispensable.

For *S*. *epidermidis* ET-024, a significant upregulation (p < 0.01) of genes encoding resistance to methicillin (*mecA*; 60.5 fold), aminoglycosides (*aacA*; 104.2 fold) and erythromycin (*ermA-1*, *ermA-2*, *ermA-3*; 219.7 to 269.5 fold) was observed in dual species biofilms compared to monospecies biofilms. Six of the 7 *S*. *epidermidis* ET-024 urease genes (*ureABCDEF*) were significantly downregulated (-2.2 to -2.7 fold) in dual species biofilms. Other significantly downregulated genes in dual species biofilms included genes encoding transporters (*lacEF*, -2.5 to -3.7 fold; *arcD*, -2.6 fold), genes encoding proteins involved in metabolism (*lacABCD*,-2.8 to -3.4 fold) and various other genes (encoding e.g. phenol soluble modulins and proteases) (Table 1).

For *S*. *aureus* Mu50, genes encoding virulence-associated proteins were significantly (p < 0.01) upregulated in dual species biofilms compared to monospecies biofilms: *spa* (3.9 fold), *splF* (2.4 fold) and *dps* (2.4 fold). All *S*. *aureus* Mu50 urease genes were significantly downregulated (-2.0 to -6.0 fold) in dual species biofilms compared to monospecies biofilms. Also, *S*. *aureus* Mu50 genes encoding transporters (e.g. *lacF*, -2.6 fold; *ulaA*, -2.9 fold), genes encoding proteins involved in metabolism (e.g. *purH*, -3.8 fold) and genes encoding factors involved in translation were significantly less expressed in dual species biofilms compared to monospecies biofilms.

Subsequently we set out experiments to confirm these RNA-Seq based observations with qPCR. Prior to qPCR analyses, suitable reference genes for normalization were selected. For *S*. *aureus* Mu50, the expression of reference genes *fabD*, *gyrA*, *proC*, *pyk*, *rho* and *tpi* was evaluated. Based on initial testing, reference genes *fabD* and *rho* were selected and their gene expression values were used to normalize the expression values of *S*. *aureus* Mu50 urease genes (Fig A in S1 File). For *S*. *epidermidis* ET-024, the expression of 7 potential reference genes was investigated (Fig B in S1 File). Based on initial testing, reference genes *aroE*, *gmk*, *folA* and *hsp60* were selected and their expression values were used to normalize the expression values of *S*. *epidermidis* ET-024 urease genes. For both strains, RNA-Seq analysis confirmed that the expression of the selected reference genes did not differ between single and dual species biofilms.

**Fig 2:**
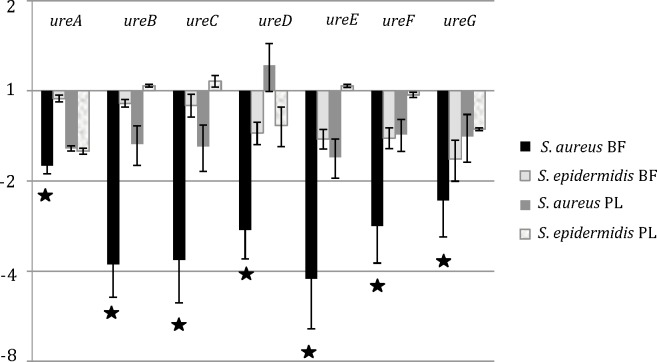
The fold change (determined by qPCR) in urease gene expression of *S*. *aureus* Mu50 and *S*. *epidermidis* ET-024 in dual species biofilms and dual species planktonic cultures compared to monospecies biofilms (BF) and monospecies cultures (PL) (normalized by the expression values of selected reference genes), respectively. Three biological replicates were included and each experiment was repeated twice per biological replicate. SEM (error bars) was calculated for all conditions. *: p < 0.01. BF: biofilm; PL: planktonic.

There was significantly less expression of *ureABCDEFG* genes of *S*. *aureus* Mu50 in dual species biofilms compared to monospecies biofilms and the fold change varied from—1.7 (*ureA*) to -4.2 (*ureE*) (Fig 2); this difference is apparently biofilm-specific, as there were no significant differences in urease gene expression of *S*. *aureus* Mu50 in dual species planktonic cultures compared to a *S*. *aureus* Mu50 monocultures (Fig 2).

For *S*. *epidermidis* ET-024, there were no significant differences in the expression of the urease genes in dual species biofilms compared to monospecies biofilms and the same was true for planktonic cultures (Fig 2).

### Urease activity in single and dual species biofilms

**Fig 3:**
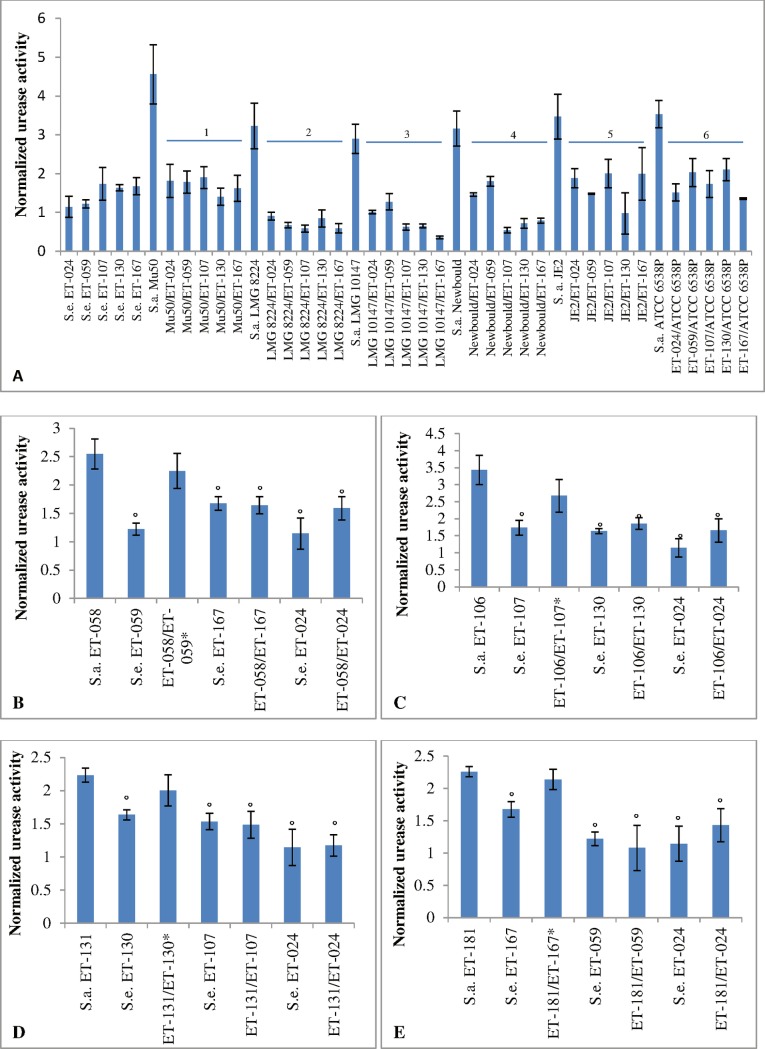
The urease activity in single and dual species biofilms of *S*. *aureus* and *S*. *epidermidis* (units urease/L normalized to the number (log_10_) of CFU per biofilm). Three biological replicates were included and each experiment was repeated at least 6 times per biological replicate. Standard error mean (SEM; error bars) was calculated for all conditions. **A**: The urease activity in single and dual species biofilms formed by *S*. *epidermidis* ET isolates (ET-024, ET-059, ET-107, ET-130 and ET-167) and *S*. *aureus* reference strains (Mu50, LMG 8224, LMG 10147, Newbould605 and ATCC 6568P). 1: p < 0.01, compared to *S*. *aureus* Mu50; 2: p < 0.01, compared to *S*. *aureus* LMG 8224; 3: p < 0.01, compared to *S*. *aureus* LMG 10147; 4: p < 0.01, compared to *S*. *aureus* Newbould; 5: p < 0.01, compared to *S*. *aureus* JE2; 6: p < 0.01, compared to *S*. *aureus* ATCC 6538P. **B-E**: The urease activity in single and dual species biofilms formed by *S*. *aureus* ET isolates (ET-058, ET-106, ET-131 and ET-181) and *S*. *epidermidis* ET isolates (ET-024, ET-059, ET-107, ET-130 and ET-167). *: both *S*. *epidermidis* and *S*. *aureus* isolates were obtained from the same ET biofilm. °; p < 0.01, compared to *S*. *aureus* ET-058 (Fig 1B), *S*. *aureus* ET-106 (Fig 1C), *S*. *aureus* ET-131 (Fig 1D) and *S*. *aureus* ET-181 (Fig 1E), respectively.

In order to further substantiate the results of the transcriptomics analysis, urease activity in single and dual species biofilms was determined. In order to allow comparing data between different biofilms, the urease activity (units urease/L) was normalized to the number of CFU per biofilm (Table D in S1 File). After 24 hours of incubation, urease activity in dual species biofilms of *S*. *aureus* Mu50 and *S*. *epidermidis* ET-024 biofilms was significantly lower than the activity in *S*. *aureus* Mu50 biofilms but similar to the activity in *S*. *epidermidis* ET-024 monospecies biofilms (Fig 3A). Also, urease activity in dual species biofilms formed by *S*. *epidermidis* ET isolates (i.e. ET-024, ET-059, ET-107, ET-130 and ET-167) and *S*. *aureus* reference strains (i.e. Mu50, LMG 8224, LMG 10147, Newbould 305, JE2 and ATCC 6538P) was significantly lower than the activity in monospecies biofilms formed by *S*. *aureus* reference strains (Fig 3A). However, urease activity in dual species biofilms of *S*. *epidermidis* ET isolates and *S*. *aureus* reference strains was similar to urease activity in monospecies biofilms of *S*. *epidermidis* ET isolates. Also, urease activity in biofilms formed by various other *S*. *aureus* (ET-058, ET-106, ET-131 and ET-181) and *S*. *epidermidis* (ET-059, ET-107, ET-130 and ET-167) isolates, all obtained from ET biofilms, was investigated (Fig 3B–3E). Urease activity was significantly lower in dual species biofilms formed by *S*. *aureus* ET isolates and *S*. *epidermidis* ET isolates compared to the activity in biofilms formed by *S*. *aureus* ET isolates. However, this effect was not observed when *S*. *aureus* and *S*. *epidermidis* isolates originated from the same ET biofilm, e.g. the urease activity in dual species biofilms formed by *S*. *aureus* ET-058 and *S*. *epidermidis* ET-059 was similar to the urease activity in *S*. *aureus* ET-058 monospecies biofilms (Fig 3B). Also, the urease activity in *S*. *epidermidis* ET monospecies biofilms and dual species biofilms formed by *S*. *aureus* ET isolates and *S*. *epidermidis* ET isolates is similar.

### The effect of antibiotics on single and dual species biofilms of *S*. *aureus* Mu50 and *S*. *epidermidis* ET-024

RNA-Seq analysis showed that the *S*. *epidermidis* ET-024 genes encoding oxacillin, erythromycin and tobramycin resistance were significantly upregulated in dual species biofilms. Therefore, we exposed single and dual species biofilms of *S*. *aureus* Mu50 and *S*. *epidermidis* ET-024 to these antibiotics. Prior to the experiments, the MIC of oxacillin, erythromycin and tobramycin for S. *aureus* Mu50 and S. *epidermidis* ET-024 was determined. For *S*. *epidermidis* ET-024, the MIC of oxacillin was 0.5 μg/ml, the MIC of erythromycin was 0.25 μg/ml and the MIC of tobramycin was 2 μg/ml. The MICs for *S*. *aureus* Mu50 were higher: MIC of oxacillin was > 64 μg/ml, MIC of erythromycin was > 128 μg/ml and MIC of tobramycin was 128 μg/ml.

**Fig 4:**
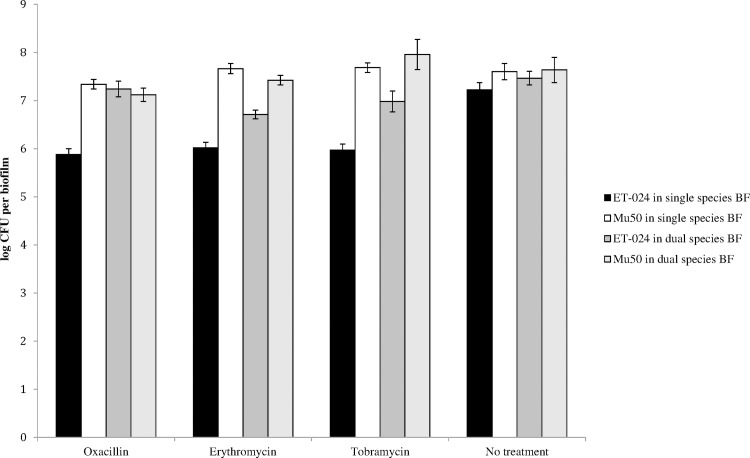
The number of *S*. *epidermidis* ET-024 and *S*. *aureus* Mu50 cells (log_10_ CFU/biofilm) in single and dual species biofilms after oxacillin (4 μg/ml), erythromycin (2 μg/ml) and tobramycin (16 μg/ml) treatments. Three biological replicates were included and each experiment was repeated twice per biological replicate. SEM (error bars) was calculated for all conditions.

Single and dual species biofilms of *S*. *epidermidis* ET-024 and *S*. *aureus* Mu50 were exposed to antibiotics for 20 hours, after 4 hours of incubation. The number of CFU/biofilm recovered after treatment did not differ between dual species biofilms and *S*. *aureus* Mu50 biofilms when biofilms were exposed to the antibiotics tested (Fig 4). In contrast, significantly more *S*. *epidermidis* ET-024 cells survived treatment when in a dual species biofilm than when in a monospecies biofilm (Fig 4).

### Virulence in a *C*. *elegans* infection model

Prior to the experiments, different test conditions were evaluated (S1 File).We evaluated the survival of nematodes when infected with *S*. *aureus* Mu50 *S*. *aureus* JE2 (the wild-type strain used for creating the NARSA mutants) and observed no significant difference in survival (48 hours p.i.) (Table 2). Similarly, survival rates were comparable following infection with *S*. *aureus* Mu50 and *S*. *epidermidis* ET-024 on the one hand and *S*. *aureus* JE2 and *S*. *epidermidis* ET-024 on the other.

**Table 2:** Survival (expressed as percentage of uninfected control) of *C*. *elegans* after infection (48 hours p.i.) with *S*. *epidermidis* and *S*. *aureus* strains. The CFU(log_10_) per nematode after infection is shown.

Infected with	% survival*	Log CFU/nematode
*S*. *aureus* Mu50	63.3 ± 6.3	4.21 ± 0.24
*S*. *aureus* JE2	67.3 ± 7.7	4.63 ± 0.18
*S*. *epidermidis* ET-024	81.9 ± 3.2	3.58 ± 0.18
NE286 (lacking functional *spa*)	80.3 ± 3.8	4.79 ± 0.19
NE1764 (lacking functional *splF*)	82.2 ± 3.6	4.75 ± 0.20
NE1969 (lacking functional *dps*)	89.1 ± 2.2	4.95 ± 0.18
*S*. *aureus* Mu50 + *S*. *epidermidis* ET-024	49.2 ± 7.7	4.21 ± 0.21 (*S*. *aureus*)/ 3.68 ± 0.15 (*S*. *epidermidis*)
*S*. *aureus* JE2 + *S*. *epidermidis* ET-024	49.8 ± 6.6	4.21 ± 0.19 (*S*. *aureus*)/ 3.68 ± 0.14 (*S*. *epidermidis*)
NE286 + *S*. *epidermidis* ET-024	85.3 ± 8.6	4.23 ± 0.13 (*S*. *aureus*)/ 4.02 ± 0.17 (*S*. *epidermidis*)
NE1764 + *S*. *epidermidis* ET-024	86.3 ± 8.6	4.24 ± 0.24 (*S*. *aureus*)/ 4.05 ± 0.24 (*S*. *epidermidis*)
NE1969 + *S*. *epidermidis* ET-024	90.3 ± 6.3	4.28 ± 0.11 (*S*. *aureus*)/ 3.84 ± 0.21 (*S*. *epidermidis*)

*: compared to uninfected control

*C*. *elegans* were subsequently infected with NARSA mutants (lacking functional *spa*, *splF* or *dps* genes) together with *S*. *epidermidis* ET-024 and a significant increase in survival was observed compared to co-infection with *S*. *aureus* JE2 and *S*. *epidermidis* ET-024 (Table 2).

We also determined the number of staphylococcal CFU/nematode after (co-)infection; no significant differences (p > 0.01) in number of CFU/nematode were observed in the different test conditions (Table 2).

## Discussion

Both *S*. *aureus* and *S*. *epidermidis* are often isolated from biofilm infections that are related to the presence of indwelling medical devices [24–26]. Different interactions between these two species have been described [39,44] and in the present study we investigated the mutual effect of *S*. *epidermidis* and *S*. *aureus* in dual species biofilms.

Our results suggest that the presence of *S*. *epidermidis* in dual species biofilms with *S*. *aureus* leads to slow-down of metabolism. Using a resazurin-based assay, we observed significantly less metabolic activity in dual species biofilms of *S*. *aureus* and *S*. *epidermidis* compared to *S*. *aureus* monospecies biofilms. Subsequently, lesser acids are produced; the pH in dual species biofilms was significantly higher than in *S*. *aureus* Mu50 monospecies biofilms, while the pH of dual species biofilms and *S*. *epidermidis* ET-024 biofilms were virtually identical (after 24 hours of incubation). So, there is less need for urease activity to balance the low pH. The transcriptome analysis showed that all *S*. *aureus* Mu50 urease genes were significantly downregulated in dual species biofilms compared to monospecies Mu50 biofilms. Also, there was lower urease activity in mixed biofilms. For *S*. *epidermidis* ET-024, gene expression data (qPCR) indicate that there is no significant difference in urease gene expression in dual species biofilms compared to monospecies biofilms. This effect was not observed with *S*. *epidermidis* and *S*. *aureus* strains isolated from the same clinical sample, which suggests that these are somehow “adapted” to each other. The mechanism underlying this adaptation is unknown and will require further investigation. The effect of *S*. *epidermidis* on *S*. *aureus* was biofilm specific, as no significant changes in urease and metabolic activity were observed in dual species planktonic cultures. The exact mechanism by which *S*. *epidermidis* ET-024 influences the metabolic activity of *S*. *aureus* Mu50 in dual species biofilms is still unclear. However, the inhibitory effect of a less virulent commensal bacterium (e.g. *S*. *epidermidis* ET-024) on the metabolism of a more virulent bacterium (e.g. *S*. *aureus* Mu50) in dual species biofilms may contribute to the so-called colonization resistance [45].

RNA-Seq data showed no significant differences in expression levels of genes involved in biofilm formation, suggesting that the underlying mechanisms of biofilm formation are not different between single- and dual-species biofilms. RNA-Seq did show a significant upregulation of *S*. *epidermidis* ET-024 genes encoding resistance to oxacillin, erythromycin and tobramycin in dual species biofilms compared to monospecies biofilms. To investigate this, we exposed biofilms to antibiotics in a concentration of 8 X the lowest MIC value (i.e. the MIC for *S*. *epidermidis* ET-024). After treating dual species biofilms with oxacillin, erythromycin or tobramycin, significantly more *S*. *epidermidis* ET-024 cells survived in dual species biofilms, compared to monospecies biofilms, confirming that *S*. *epidermidis* ET-024 is less susceptible to antibiotics in a dual species biofilm than in a monospecies biofilm. It is tempting to suggest that dual species biofilm infections are harder to treat than single species. Regardless, the mechanism(s) involved remain to be elucidated.

For *S*. *aureus* Mu50, we observed a significant upregulation of virulence-associated genes (*spa*, *splF* and *dps*) in dual species biofilms and this was further examined using a *C*. *elegans* infection assay. This model system is well-suited to investigate the contribution of specific gene products to virulence [46,47]. NARSA transposon mutants lacking a functional *spa*, *splF* or *dps* gene were used to infect *C*. *elegans*. The survival of *C*. *elegans* significantly increased when nematodes were infected with *S*. *epidermidis* ET-024 together with NE286 (lacking functional *spa*), NE1764 (lacking functional *splF*) or NE1929 (lacking functional *dps*) compared to infection with *S*. *epidermidis* ET-024 and wild-type *S*. *aureus* JE2.

Our results demonstrated that in dual species biofilms of *S*. *aureus* Mu50 and *S*. *epidermidis* ET-024 genes involved in metabolism, urease activity, antibiotic resistance and virulence are differently expressed compared to monospecies biofilms, and that these differences in expression lead to profound differences in physiology.

A better understanding of these interactions could be key to develop more effective approaches to treat these dual species biofilm infections.

## Supporting information

S1 File All supplementary data, including data on expression stability of reference genes for qPCR, biofilm forming capacities of all strains investigated, and results of preliminary tests with *C. elegans*. (DOCX)
