# Supplementary material for: Metabolic activity, urease production, antibiotic resistance and virulence in dual species biofilms of Staphylococcus epidermidis and Staphylococcus aureus
Source: PLoS One. 2017 Mar 6;12(3):e0172700. doi: 10.1371/journal.pone.0172700 (PMC5338783; doi:10.1371/journal.pone.0172700)
Supplement: S1 File — (DOCX) [file pone.0172700.s001.docx]

**Supplementary data:**

**A. Investigating the expression stability of the reference genes of *S*. *epidermidis* ET-024 and *S*. *aureus* Mu50.**

Species-specific primers targeting the urease genes of *S*. *aureus* Mu50 and *S*. *epidermidis* ET-024 were developed using the NCBI primer design tool ([www.ncbi.nlm.nih.gov/tools/primer-blast/](http://www.ncbi.nlm.nih.gov/tools/primer-blast/)). Primers were selected by blasting the primer sequences towards *S*. *aureus* Mu50 and *S*. *epidermidis* ET-024 genomeswww.ncbi.nlm.nih.gov/BLAST ([www.ncbi.nlm.nih.gov/BLAST/](http://www.ncbi.nlm.nih.gov/BLAST/)). Hairpin formation and dimerization was checked using the oligocalc tool ([www.basic.northwestern.edu/biotools/oligocalc.html](http://www.basic.northwestern.edu/biotools/oligocalc.html)) and primer pairs with hairpin formation and/or dimerization were excluded.

For *S*. *epidermidis* ET-024, the expression of 7 reference genes was tested. Based on qPCR data (Cq values and melting curves) and on the results from the GeNorm (BioRad) software, reference genes *aroE*, *gmk*, *folA* and *hsp60* were selected and their expression values were used to normalize the expression values of *S*. *epidermidis* ET-024 urease genes (Table S1). Also, the specificity of the primers targeting the urease genes of *S*. *epidermidis* ET-024 was checked. There was no difference between the Cq-values of the qPCR reactions performed on *S*. *epidermidis* ET-024 cDNA and *S*. *epidermidis* ET-024 cDNA mixed with *S*. *aureus* Mu50 cDNA. Also, the shape of melting curves of these qPCR reactions was very similar. So, the primers specifically target the *S*. *epidermidis* ET-024 urease genes (Table S1).

Primers were tested for S. aureus Mu50 reference genes *fabD*, *gyrA*, *proC*, *pyk*, *rho* and *tpi*. Based on qPCR data (Cq values and melting curves) and on the results of the GeNorm software, reference genes *fabD* and *rho* of *S*. *aureus* Mu50 were selected and their gene expression values were used to normalize the expression values of the *S*. *aureus* Mu50 urease genes . Next, the specificity of the primers targeting the urease genes of *S*. *aureus* Mu50 was checked. There was no difference between the Cq-values of the qPCR reactions performed on *S*. *aureus* Mu50 cDNA and *S*. *aureus* Mu50 cDNA mixed with *S*. *epidermidis* ET-024 cDNA and also, the shape of melting curves of the qPCR reactions was very similar. So, the designed primers specifically target the *S*. *aureus* Mu50 urease genes (Table S1).

**Figure legends**: The graphs show the stability of the different combinations of reference genes. The combination with the smallest numbers of reference genes that has a v-value (as calculated by the GeNorm software) smaller than 0.15, was selected to be included in the experiments.

**Figure** **A**: The expression stability of the different reference genes of *S*. *epidermidis* ET-024.


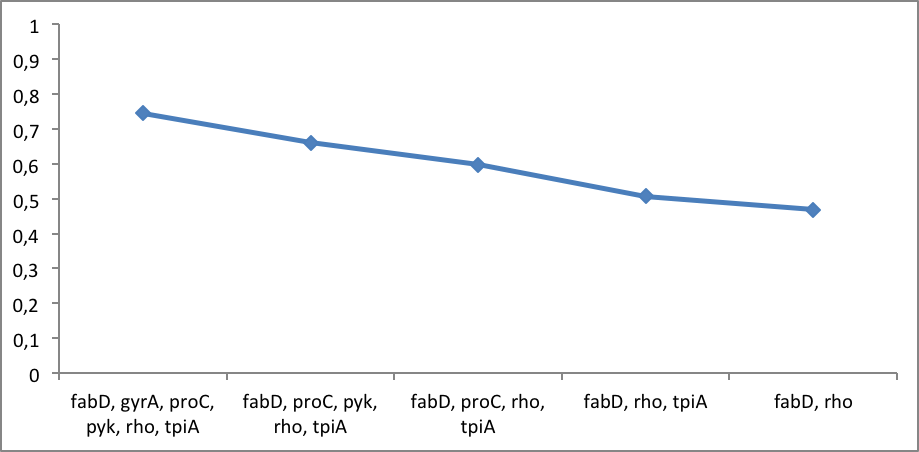


v=0.09

v=0.12

v=0.13

**Figure** **B**: The expression stability of the different reference genes of *S*. *aureus* Mu50.


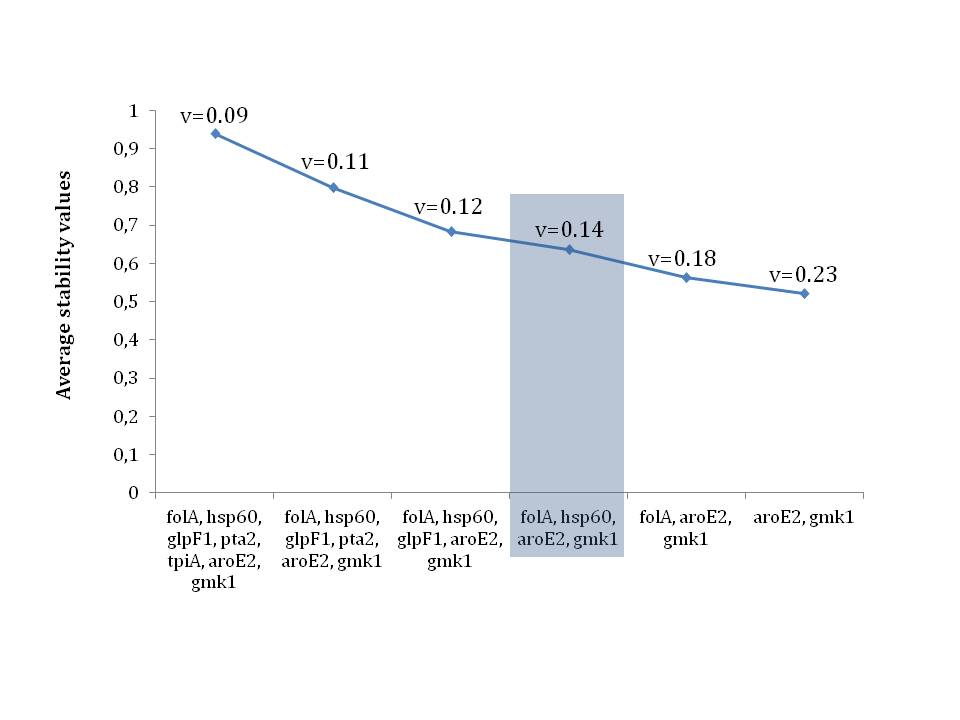


**Species-specific primers used in the present study**

|  | **Forward primer sequence** | **Reverse primer sequence** |
| --- | --- | --- |
| *S. aureus* Mu50 |  |  |
| *ureA* | ggt gca cgc gat ggt aag | atc atg tgt tcg aca cca tcc |
| *ureB* | ttt cgt ggt atg gtc aat ggt c | cac gtt ttc agc acc gtt atc |
| *ureC* | tgc cgc tga agg taa aat cg | aat gcg acc tct gct tgt tc |
| *ureD* | act ctc att atg gct ctg ctt ac | acc tgc aaa gcc atg tgt agg |
| *ureE* | tcc aga aaa aca aaa gca cgt cg | gcc atg gtc tgt aac gac tc |
| *ureF* | tcc aac agg tgc ttt cag tc | aaa cgc atg gct aaa cca tca |
| *ureG* | tac tgc gat tcg tga aga tgc | ctg cta agt ata cgc cac cag |
| *fabD* | CCT TTA GCA GTA TCT GGA CC | GAA ACT TAG CAT CAC GCC |
| *rho* | GGA AGA TAC GAC GTT CAG AC | GAA GCG GGT GGA AGT TTA |
| *S. epidermidis* ET-024 |  |  |
| *ureA* | acg tcg tag aaa agc aag agg | aag ttc agc tac cgt ttt acc at |
| *ureB* | ata cgg gcg ata gac cta tac aag | ata tcg cca tcg act aaa cca c |
| *ureC* | aaa gaa caa gcg gga cca c | tgc cac ctt taa caa caa gat cg |
| *ureD* | ctt gac ggc att ggg tat atg g | agt caa aat acg aac ggc caa t |
| *ureE* | taa agg aaa tgg gag aca ttg ct | tca gtt tct gta aat tgt gca ggt |
| *ureF* | gcg cta agc aaa tgg gta ca | aag cac aca gca gga tga ag |
| *ureG* | tgt tgg tgc agg taa aac cc | gga caa cct cca gtt tcc ac |
| *folA* | GGG AAA CCA TTG CCA AAT AGA C | CGA ATA ACG TTT GTC CTC CAA ATA |
| *hsp60* | TCT TAA GAA TGT TAC AAG TGG TGC AA | AAT CTC ATG GAG CGC TTC TAT AGC |
| *aroE* | TGG AAA ACA CGT ATG AAG CGA | TTA CCG CCC CAA CAG ATT TC |
| *gmk* | gcc ctt cag gtg ttg gaa ag | cca tca att tca cct tca cgc |

**Table A** : Species-specific primers targeting the urease genes and selected reference genes of *S*. *aureus*  Mu50 and *S*. *epidermidis* ET-024

**B. Number (log_10_) of CFU per biofilm and evaluating the biofilm forming capacities of all strains investigated in this study.**

Prior to all experiments, we have investigated the relative fitness of *S*. *aureus* and *S*. *epidermidis* in dual species biofilms when inoculated at different ratios (i.e. 1/1, 1/10 and 10/1). After incubation, cells were removed from the wells and serial dilutions (10^-1^ to 10^-9^) were made. The MHA plates were incubated at 37°C until a morphological difference in colony appearance was observed (48 to 72 hours). *S*. *aureus* colonies were larger and goldisch while *S*. *epidermidis* colonies are smaller and white. The number of *S*. *aureus* and *S*. *epidermidis* CFU in dual species biofilms was determined by counting the larger, goldisch colonies and the smaller white colonies, respectively.

The results showed that the different ratios of both species are still the same compared to inoculation ratios. So, there is no effect of one species on the survival of the other species (Table S2).

|  | **Start** (log_10_ CFU/BF) | **After 24 hours** (log_10_ CFU/BF)  *Mu50* *ET-024* |
| --- | --- | --- |
| *S*. *aureus* Mu50 (1) | 6.66 + 0.14 | 8.12 + 0.83 |
| *S*. *epidermidis* ET-024 (1) | 6.75 + 0.08 | 8.72 + 0.49 |
| *S*. *aureus* Mu50 (10) | 7.74 + 0.09 | 9.19 + 1.01 |
| *S*. *epidermidis* ET-024 (10) | 7.68 + 0.10 | 9.97 + 0.87 |
| *S*. *aureus* Mu50 (1*)*/ *S*. *epidermidis* ET-024 (1) | 6.63 + 0.13 / 6.72 + 0.09 | 7.84 + 0.69 8.01 + 0.55 |
| *S*. *aureus* Mu50 (10)/ *S*. *epidermidis* ET-024 (1) | 7.77 + 0.08 / 6.69 + 0.07 | 8.98 + 0.74 8.03 + 0.39 |
| *S*. *aureus* Mu50 (1)/ *S*. *epidermidis* ET-024 (10) | 6.66 + 0.12 / 7.68 + 0.06 | 7.91 + 0.76 9.15 + 0.46 |

**Table B**: Number (log_10_) of CFU in dual species biofilms when *S*. *aureus* Mu50 and *S*. *epidermidis* ET-024 were inoculated at different ratios

In addition, the biofilm forming capacities of all strains investigated in this study were evaluated by a crystal violet assay. The results demonstrated that all strains were able to form biofilms and the strongest biofilm forming strains are the *S*. *aureus* reference strains (i.e. Mu50, LMG8224, LMG10147, Newbould and ATCC6538) (Table S3).

|  | average Abs (590 nm) | SEM |
| --- | --- | --- |
| *S*. *aureus* ET-058 | 2.26 | 0.19 |
| *S*. *aureus* ET-106 | 2.37 | 0.15 |
| *S*. *aureus* ET-137 | 2.11 | 0.17 |
| *S*. *aureus* ET-181 | 2.99 | 0.13 |
| *S*. *aureus* Mu50 | 4.43 | 0.15 |
| *S*. *aureus* LMG8224 | 3.85 | 0.12 |
| *S*. *aureus* LMG10147 | 3.60 | 0.20 |
| *S*. *aureus* Newbould | 3.20 | 0.12 |
| *S*. *aureus* ATCC6538 | 3.37 | 0.16 |
| *S*. *epidermidis* ET-024 | 2.45 | 0.15 |
| *S*. *epidermidis* ET-059 | 2.67 | 0.13 |
| *S*. *epidermidis* ET-107 | 2.52 | 0.14 |
| *S*. *epidermidis* ET-130 | 2.08 | 0.10 |
| *S*. *epidermidis* ET-167 | 2.34 | 0.16 |

**Table C:** Results of the crystal violet of all *S*. *aureus* and *S*. *epidermidis* strains investigated in this study.

In order to determine the number of *S*. *epidermidis* and *S*. *aureus* CFU per biofilm, dual species biofilms of both species were inoculated (ratio 1/1) (Table S4).

|  | **Total CFU(log_10_)/ biofilm** | **S. *aureus* CFU (log_10_) in dual species biofilms** | ***S*. *epidermidis* CFU (log_10_) in dual species biofilms** |
| --- | --- | --- | --- |
| *S*. *aureus* ET-058 | 8.14 + 0.26 |  |  |
| *S*. *aureus* ET-106 | 7.64 + 0.46 |  |  |
| *S*. *aureus* ET-131 | 7.98 + 0.50 |  |  |
| *S*. *aureus* ET-181 | 8.09 +1.01 |  |  |
| S. *aureus* Mu50 | 8.04 + 0.96 |  |  |
| *S*. *aureus* LMG 8224 | 8.66 +1.24 |  |  |
| *S*. *aureus* LMG 10147 | 9.13 +1.39 |  |  |
| *S*. *aureus* Newbould | 8.92 + 0.85 |  |  |
| *S*. *aureus* ATCC 6538 | 8.86 + 0.23 |  |  |
| *S*. *epidermidis* ET-024 | 8.68 + 0.33 |  |  |
| *S*. *epidermidis* ET-059 | 8.49 + 0.61 |  |  |
| *S*. *epidermidis* ET-107 | 6.42 + 0.19 |  |  |
| *S*. *epidermidis* ET-130 | 8.33 + 0.45 |  |  |
| *S*. *epidermidis* ET-167 | 8.44 + 0.34 |  |  |
| *S*. *aureus* ET-058/*S*. *epidermidis* ET-059 | 7.94 + 0.47 | 7.67 + 0.33 | 7.60 + 0.41 |
| *S*. *aureus* ET-058/*S*. *epidermidis* ET-167 | 8.01 + 0.82 | 7.75 + 0.66 | 7.66 + 0.53 |
| *S*. *aureus* ET-058/*S*. *epidermidis* ET-024 | 7.92 + 0.29 | 7.59 + 0.13 | 7.64 + 0.18 |
| *S*. *aureus* ET-106/*S*. *epidermidis* ET-107 | 7.79 + 0.13 | 7.46 + 0.07 | 7.51 + 0.08 |
| *S*. *aureus* ET-106/*S*. *epidermidis* ET-130 | 7.91 + 0.27 | 7.68 + 0.19 | 7.52 +0.21 |
| *S*. *aureus* ET-106/*S*. *epidermidis* ET-024 | 7.89 + 0.67 | 7.67 + 0.49 | 7.49 + 0.52 |
| *S*. *aureus* ET-131/*S*. *epidermidis* ET-130 | 7.85 + 0.58 | 7.62 + 0.29 | 7.46 + 0.47 |
| *S*. *aureus* ET-131/*S*. *epidermidis* ET-107 | 8.01 + 0.46 | 7.78 + 0.17 | 7.62 + 0.31 |
| *S*. *aureus* ET-131/*S*. *epidermidis* ET-024 | 7.61 + 0.41 | 7.28 + 0.23 | 7.34 + 0.30 |
| *S*. *aureus* ET-181/*S*. *epidermidis* ET-167 | 7.86 + 0.39 | 7.71 + 0.32 | 7.33 + 0.29 |
| *S*. *aureus* ET-181/*S*. *epidermidis* ET-059 | 7.77 + 0.68 | 7.59 + 0.51 | 7.3 + 0.58 |
| *S*. *aureus* ET-181/*S*. *epidermidis* ET-024 | 7.67 + 0.59 | 7.47 + 0.47 | 7.23 + 0.51 |
| *S*. *aureus* Mu50/*S*. *epidermidis* ET-059 | 8.04 + 0.62 | 7.83 + 0.27 | 7.61 + 0.54 |
| *S*. *aureus* Mu50/*S*. *epidermidis* ET-107 | 8.19 + 0.11 | 7.97 + 0.04 | 7.79 + 0.07 |
| *S*. *aureus* Mu50/*S*. *epidermidis* ET-130 | 7.98 + 0.09 | 7.61 + 0.05 | 7.74 + 0.07 |
| *S*. *aureus* Mu50/*S*. *epidermidis* ET-167 | 7.83 + 0.22 | 7.64 + 0.14 | 7.37 + 0.18 |
| *S*. *aureus* Mu50/*S*. *epidermidis* ET-024 | 8.24 + 0.78 | 7.89 + 0.54 | 7.98 + 0.71 |
| *S*. *aureus* LMG 8224/*S*. *epidermidis* ET-024 | 8.84 + 0.23 | 8.43 + 0.15 | 8.11 + 0.19 |
| *S*. *aureus* LMG 8224/*S*. *epidermidis* ET-059 | 8.80 + 0.69 | 8.55 + 0.41 | 8.44 + 0.55 |
| *S*. *aureus* LMG 8224/*S*. *epidermidis* ET-107 | 8.91 + 0.62 | 8.73 + 0.39 | 8.44 + 0.59 |
| *S*. *aureus* LMG 8224/*S*. *epidermidis* ET-130 | 8.81 + 0.51 | 8.29 + 0.40 | 8.65 + 0.43 |
| *S*. *aureus* LMG 8224/*S*. *epidermidis* ET-167 | 8.96 + 0.74 | 8.81 + 0.64 | 8.43 + 0.68 |
| *S*. *aureus* LMG 10147/*S*. *epidermidis* ET-024 | 8.77 + 0.43 | 8.57 + 0.28 | 8.33 + 0.37 |
| *S*. *aureus* LMG 10147/*S*. *epidermidis* ET-059 | 8.58 + 0.37 | 8.40 + 0.25 | 8.11 + 0.29 |
| *S*. *aureus* LMG 10147/*S*. *epidermidis* ET-107 | 8.94 + 0.48 | 8.83 + 0.41 | 8.24 + 0.35 |
| *S*. *aureus* LMG 10147/*S*. *epidermidis* ET-130 | 8.71 + 0.37 | 8.51 + 0.24 | 8.28 + 0.32 |
| *S*. *aureus* LMG 10147/*S*. *epidermidis* ET-167 | 8.84 + 0.24 | 8.37 + 0.17 | 8.66 + 0.21 |
| *S*. *aureus* Newbould/*S*. *epidermidis* ET-024 | 8.75 + 0.68 | 8.41 + 0.57 | 8.48 + 0.53 |
| *S*. *aureus* Newbould/*S*. *epidermidis* ET-059 | 8.62 + 0.61 | 8.15 + 0.43 | 8.44 + 0.55 |
| *S*. *aureus* Newbould/*S*. *epidermidis* ET-107 | 8.75 + 0.52 | 8.32 + 0.39 | 8.55 + 0.47 |
| *S*. *aureus* Newbould/*S*. *epidermidis* ET-130 | 8.92 + 0.48 | 8.74 + 0.31 | 8.46 + 0.38 |
| *S*. *aureus* Newbould/*S*. *epidermidis* ET-167 | 8.73 + 0.37 | 8.58 + 0.13 | 8.18 + 0.31 |
| *S*. *aureus* ATCC 6538/*S*. *epidermidis* ET-024 | 8.63 + 0.66 | 8.46 + 0.41 | 8.14 + 0.55 |
| *S*. *aureus* ATCC 6538/*S*. *epidermidis* ET-059 | 8.55 + 0.54 | 8.40 + 0.45 | 8.02 + 0.15 |
| *S*. *aureus* ATCC 6538/*S*. *epidermidis* ET-107 | 9.28 + 0.21 | 9.10 + 0.15 | 8.80 + 0.07 |
| *S*. *aureus* ATCC 6538/*S*. *epidermidis* ET-130 | 8.47 + 0.08 | 8.10 + 0.05 | 8.23 + 0.02 |
| *S*. *aureus* ATCC 6538/*S*. *epidermidis* ET-167 | 8.65 + 0.18 | 8.08 + 0.11 | 8.51 +0.05 |

**Table D** : the number (log_10_) of CFU per biofilm

**C. Evaluating different tests conditions for infecting *C*. *elegans* with NARSA mutants and *S*. *epidermidis* ET-024.**

Prior to the experiment, different test conditions were evaluated. NARSA mutants lacking functional *spa* (NE286), *dps* (NE1929) and *splF* (NE1764) genes require erythromycin in the growth medium to preserve the mutations. The wild-type *S*. *aureus* strain JE2 cannot grow in the presence of 5 µg/ml erythromycin. The effect of erythromycin on the growth rate of NARSA mutants, on the survival rate of *C*. *elegans* and on the number (log_10_) of staphylococcal CFU per nematode was determined.

In addition, a PCR assay was performed in order to confirm the presence of the transposon insertions in the NARSA strains (grown in medium with and without erythromycin, respectively).

**Materials and methods:**

The growth of NARSA mutants (NE286, NE1764 and NE1929) in function of time was investigated. To do this, a 24 well MTP was used and the first row served as blanks (Mueller Hinton Broth; MHB) while the second, third and fourth rows were filled with cell suspensions of NE286, NE1764 or NE1929 (approx. 10^5^ cells). Five µg/ml erythromycin was added to the first tree columns. The absorbance at 590 nm was measured every 30 minutes during 20 hours using a MTP reader (Envision, Perkin Elmer). Afterwards, a growth curve (absorbance in function of time) was plotted and the generation time for all strains was calculated. Next, the effect of erythromycin on the survival of *C*. *elegans* infected with NARSA mutants was evaluated. All wells of a 24 well MTP were filled with *C*. *elegans* and bacterial cell suspensions of NE286, NE1704 and NE1929 were also added. A second MTP was identically filled but 5 µg/ml erythromycin was added to all wells. Also, we evaluated the effect of erythromycin on the number (log_10_) of CFU of NARSA mutants in the nematodes (48 hours p.i.). Again, two 24 MTP per experiment were used (with or without erythromycin) and they were filled as described above. Afterwards, the worms were disrupted (Brackman *et al*., 2016) and the obtained suspensions were inoculated on MHA supplemented with 7.5% NaCl (to select for staphylococci) and on MHA supplemented with 5 µg/ml erythromycin (to select for NARSA mutants). In addition, the presence of the transposon insertion was confirmed by PCR. To do this, gene-specific primers were designed that target *spa*, *dps* and *splF* genes. Also, primers (buster and upstream) targeting the inserted transposon were included in the assay; these primer sequences were downloaded from the website of the medical center of the University of Nebraska (where the NARSA transposon mutant library was constructed; app1.unmc.edu/fgx/methods.html). Combinations of gene specific primers and transposon primers were used to confirm the mutation. The lengths of the amplicons were estimated based on information present on the NARSA website and based on *in* *silico* amplification data (Table S3). NARSA strains NE286, NE1764 and NE1929 were grown (for 48 hours) in growth medium with and without erythromycin (5 µg/ml) and afterwards, DNA was extracted according to Pitcher *et* *al.* (1989). Also, *S*. *aureus* JE2 was included to confirm the presence of functional *spa*, *splF* and *dps* genes.

**Results:**

The results of the different growth tests are summarized in Table S4. The growth curves of the NARSA mutants, cultivated in medium with or without erythromycin, were identical and no significant differences in generation time were observed between the different strains (data not shown). Also, the addition of erythromycin had no significant effect (p > 0.01) on the survival of the nematodes: 98.1 + 0.8 % (growth controls without erythromycin) versus 94.6 + 1.1 % (growth controls supplemented with 5 µg/ml erythromycin). The survival rate (48 hours p.i.) of *C*. *elegans* infected with NARSA mutants was high: 80.3 + 3.8 % (NE286), 85.1 + 3.2 % (NE1764) and 83.2 + 3.2 % (NE1929). This was almost identical to the survival rate of *C*. *elegans* when erythromycin was added to the *C*. *elegans* assay: 82.9 + 2.4 % (NE286 + E), 85.2 + 3.8 % (NE1764 + E) and 92.3 + 2.3 % (NE1929 + E). After 24 hours, the number (log_10_) of CFU per worm were determined and no significant differences (p > 0.01) in the number (log_10_) of CFU between the different conditions were observed.

These results demonstrated that it is possible to infect *C*. *elegans* with NARSA mutants without the addition of erythromycin. These data show that the mutation is still present during and after infection, considering that loss of the mutation would have resulted in no growth on MHA supplemented with erythromycin (as observed with the wild-type *S*. *aureus* strain JE2).

The results of the PCR assay are shown in Figure S3. The lengths of the amplicons were estimated according to the 100 bp ladder. In summary, amplicons of the same length are obtained for the NARSA strains grown in medium with erythromycin and grown in medium lacking erythromycin. The lengths of the amplicons exactly match the lengths calculated (table S3) and thus, it can be concluded that the transposon insertion is present in *spa*, *splF* and *dps* genes.

**Table E**: Estimated amplicon length of functional and non functional *spa*, *splF* and *dps* genes (based on information present on the NARSA website and based on *in silico* amplification data); FW: forward primer; RV: reverse primer. *: Due to the high GC content of the 3’ end of the *dps* gene, it was not possible to design a RV primer targeting a sequence between the insertion and the 3’ end of the *dps* gene.

|  | **JE2** | **NE286** | **NE1764** | **NE1929** | **Primer sequences** |
| --- | --- | --- | --- | --- | --- |
| ***spa***  **FW +RV**  **RV + buster**  **FW + upstream** | 269 bp | 367 bp  498 bp |  |  | 5’ TTC AAT TCG TAA ACT AGG TGT AGG T 3’  + 5’ GTT GCG CAT CAG CTT TTG GA 3’  5’ GTT GCG CAT CAG CTT TTG GA 3’  + 5’ GCT TTT TCT AAA TGT TTT TTA AGT AAA TCA AGT AC  5’ TTC AAT TCG TAA ACT AGG TGT AGG T  + 5’ CTC GAT TCT ATT AAC AAG GG 3’ |
| ***splF***  **FW + RV**  **RV + buster**  **FW + upstream** | 487 bp |  | 262 bp  821 bp |  | 5’ AAC AAA CAG CCA AAG CCG AA 3’  + 5’CCA GAG CTA CCA GGC TGA AT 3’  5’CCA GAG CTA CCA GGC TGA AT 3’  + 5’ GCT TTT TCT AAA TGT TTT TTA AGT AAA TCA AGT AC 3’  5’ AAC AAA CAG CCA AAG CCG AA 3’  + 5’ CTC GAT TCT ATT AAC AAG GG 3’ |
| ***dps***  **FW + RV**  **RV + buster**  **FW + upstream** | 181 bp |  |  | *  662 bp | 5’ TTA GCG GTA GGA GGA AAC CC 3’  + 5’ CAT CAT CGC CAG CAT TAC CA 3’  5’ CAT CAT CGC CAG CAT TAC CA 3’  + 5’ GCT TTT TCT AAA TGT TTT TTA AGT AAA TCA AGT AC 3’  5’ TTA GCG GTA GGA GGA AAC CC 3’  + 5’ CTCGATTCTATTAACAAGGG 3’ |

**Table F**: Summary of results of the different test conditions that were evaluated prior to the *C*. *elegans* infection assays. *: inoculated on MHA + 7.5 % NaCl; **: inoculated on MHA + 5 µg/ml erythromycin.

|  | **Without erythromycin** | **Supplemented with erythromycin** |
| --- | --- | --- |
| **Generation time (min)** |  |  |
| NE286 | 49.64 + 0.67 | 48.42 + 0.80 |
| NE1764 | 45.62 + 0.30 | 49.71 + 3.49 |
| NE1929 | 51.76 + 0.55 | 50.86 + 0.64 |
|  |  |  |
| **% Survival of *C*.*elegans*** |  |  |
| No infection | 98.07 + 0.77 | 94.60 + 1.10 |
| NE286 | 80.26 + 3.81 | 82.87 + 2.44 |
| NE1764 | 82.15 + 3.62 | 85.16 + 3.75 |
| NE1929 | 89.06 + 2.19 | 92.32 + 2.28 |
|  |  |  |
| **CFU (log_10_) per nematode** |  |  |
| NE286 | 4.79 + 0.19* (4.69 + 0.04)** | 5.10 + 0.21* / 4.95 + 0.32** |
| NE1764 | 4.75 + 0.20* (4.92 + 0.16)** | 4.94 + 0.24* / 5.07 + 0.28** |
| NE1929 | 4.95 + 0.18* (4.86 + 0.15)** | 4.95 + 0.40* / 4.86 + 0.37** |

**Figure C**: Agarose gel showing the fragments after PCR amplification of the transposon insertions **A**: *spa* gene; **B**: *dps* gene; **C**: *splF* gene.

| **Lane** | **Sample** | **Length** |
| --- | --- | --- |
|  | 100 bp ladder |  |
| **1** | Blank: FW + RV | 0 bp |
| **2** | JE2: FW + RV | 269 bp |
| **3** | Blank: FW + RV | 0 bp |
| **4** | NE286: RV + buster | 367 bp |
| **5** | NE286 + erythromycin: RV + buster | 367 bp |
| **6** | Blank: RV + buster | 0 bp |
| **7** | NE286: FW + upstream | 498 bp |
| **8** | NE286 + erythromycin: FW + upstream | 498 bp |
| **9** | Blank: FW + upstream | 0 bp |

A


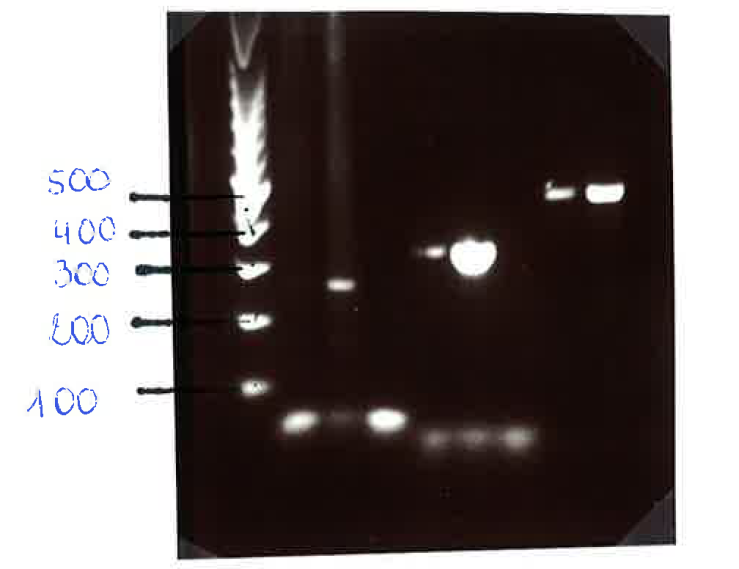


100**Average stability values**

200Reference genes of *S*. *aureus* Mu50

300

400

500

1 2 3 4 5 6 7 8 9v=0.08

| **Lane** | **Sample** | **Length** |
| --- | --- | --- |
|  | 100 bp ladder |  |
| **1** | JE2: FW + RV | 181 bp |
| **2** | JE2: FW + RV | 181 bp |
| **3** | Blank: FW + RV | 0 bp |
| **4** | NE1929: FW + upstream | 662 bp |
| **5** | NE1929 + erythromycin: FW + upstream | 662 bp |
| **6** | Blank: FW + upstream | 0 bp |

B


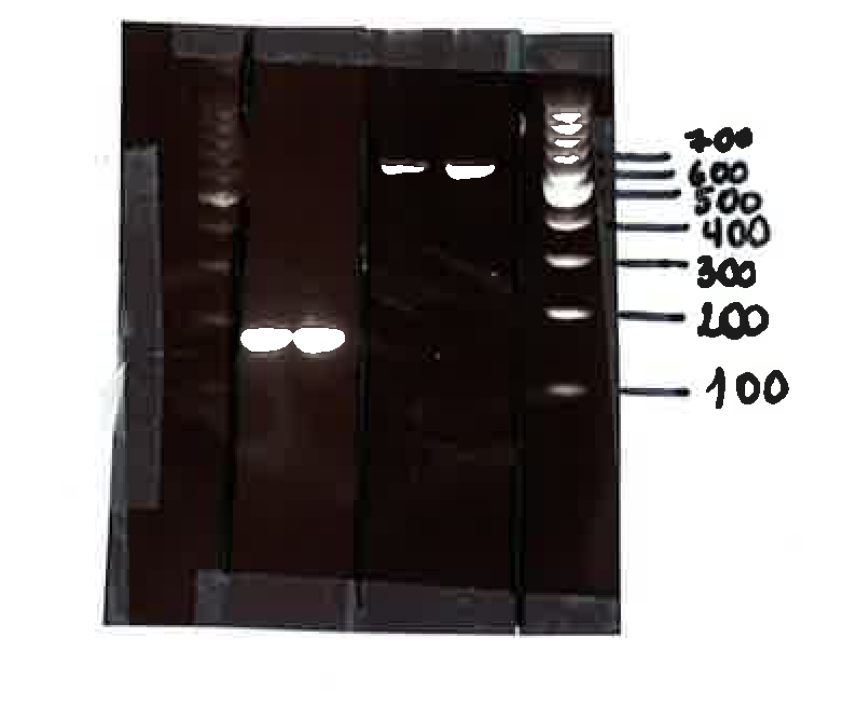


100

200

300

400

500

600

700v=0.07

1 2 3 4 5 6

| **Lane** | **Sample** | **Length** |
| --- | --- | --- |
|  | 100 bp ladder |  |
| **1** | JE2: FW + RV | 487 bp |
| **2** | JE2: FW + RV | 487 bp |
| **3** | Blank: FW + RV | 0 bp |
| **4** | NE1764: RV + buster | 262 bp |
| **5** | NE1764 + erythromycin: RV + buster | 262 bp |
| **6** | Blank: RV + buster | 0 bp |
| **7** | NE1764: FW + upstream | 821 bp |
| **8** | NE1764 + erythromycin: FW + upstream | 821 bp |
| **9** | Blank: FW + upstream | 0 bp |
|  | 100 bp ladder |  |

C


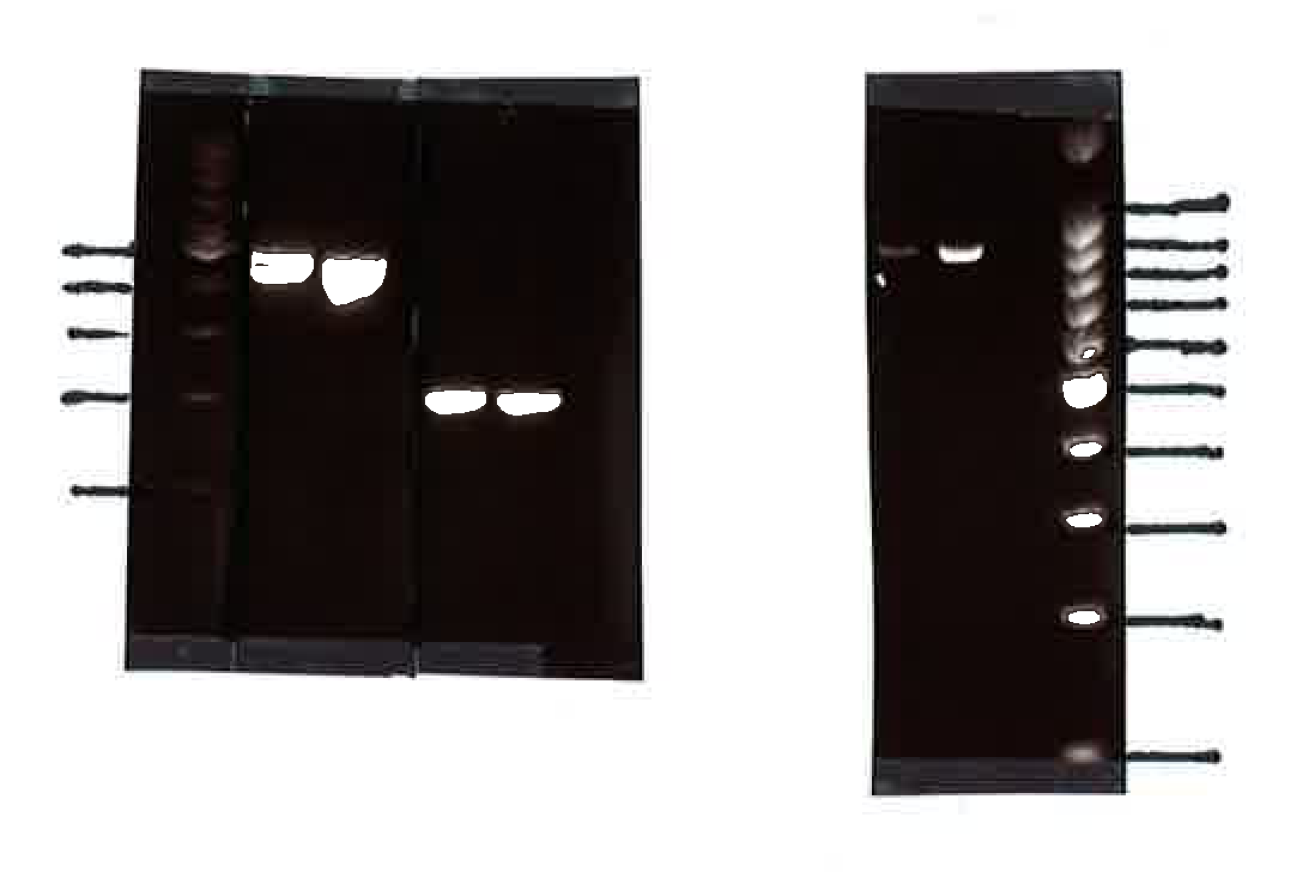


100

200

300

400

500

600

1000

900

800

700

100

200

300

400

500

1 2 3 4 5 6

7 8 9

**References:**

**Brackman G, Breyne K, De Rycke R, Vermote A, Van Nieuwerburgh F, Meyer E, et al.** **(2016)**. The Quorum Sensing Inhibitor Hamamelitannin Increases Antibiotic Susceptibility of Staphylococcus aureus Biofilms by Affecting Peptidoglycan Biosynthesis and eDNA Release. Sci Rep. 2016;6:20321.

**Pitcher, D. G., Saunders, N. A., Owen, R. J. (1989).** Rapid extraction of bacterial genomic DNA with guanidium thiocyanate. *Lett Appl Microbiol* **8**, 151-156.
